# Klotho Ameliorates Podocyte Injury through Targeting TRPC6 Channel in Diabetic Nephropathy

**DOI:** 10.1155/2022/1329380

**Published:** 2022-04-18

**Authors:** Xingmei Yao, Hengjiang Guo, Mengyao Sun, Sixuan Meng, Bingbing Zhu, Ji Fang, Jiebo Huang, Hao Wang, Lina Xing

**Affiliations:** ^1^Department of Nephrology, Putuo Hospital, Shanghai University of Traditional Chinese Medicine, Shanghai 200062, China; ^2^Department of Anesthesiology, Shanghai Children's Hospital, Shanghai Jiao Tong University, Shanghai 200062, China

## Abstract

Podocyte damage is vital for the etiopathogenesis of diabetic nephropathy (DN). Klotho (KL), a multifunctional protein, has been demonstrated to have renoprotective effects; nevertheless, the mechanism for protective effect has not been completely elucidated. Transient receptor potential cation channel subfamily C, member 6 (TRPC6), a potential target of KL, is implicated in glomerular pathophysiology. Here, we sought to determine whether KL could protect against podocyte injury through inhibiting TRPC6 in DN. We found that high glucose (HG) triggered podocyte injury as manifested by actin cytoskeleton damage along with the downregulation of KL and Synaptopodin and the upregulation of TRPC6. KL overexpression reversed HG-induced podocytes injury, whereas cotreatment with TRPC6 activator flufenamic acid (FFA) significantly abrogated the beneficial effects conferred by KL. Moreover, KL knockdown in podocytes resulted in actin cytoskeleton impairment, decreased Synaptopodin expression, and increased TRPC6 expression. In *db/db* mice, KL overexpression inhibited TRPC6 expression and attenuated diabetes-induced podocyte injury, which was accompanied by decreased albuminuria and ameliorated glomerulosclerosis. Our data provided novel mechanistic insights for KL against DN and highlighted TRPC6 as a new target for KL in podocytes to prevent DN.

## 1. Introduction

With the growing incidence and prevalence of diabetes mellitus (DM) around the world, diabetic nephropathy (DN), a severe complicating disease of DM, has been the primary cause of end-stage renal disease (ESRD) [1, 2]. Clinically, DN is characterized by progressive proteinuria which is most closely related to podocyte injury [3]. Podocytes are greatly specialised cells with intricate cell structure in glomeruli whose dysfunction results in deterioration of glomerulus filtration barrier and proteinuria [4, 5]. The precise function of podocytes relies on the proper organization of the actin cytoskeleton, and actin cytoskeletal rearrangement is a common pathological process for glomerulus diseases [4, 6]. However, the mechanisms underlying podocyte actin cytoskeleton rearrangement have not been fully clarified.

Klotho (KL), originally identified as an antiaging gene, encodes a single-pass transmembrane protein with high abundance in renal and cerebral tissues [7]. There are two distinct forms of KL. The membrane-bound form is a cofactor for fibroblast growth factor 23 (FGF23) and plays a critical role in modulating phosphate homeostasis. The secreted soluble form acts as a hormone with antioxidative stress, anti-inflammatory, and antisenescent properties [8]. Recently, the renoprotective effect of KL has aroused increasing interest. Reduced kidney KL expression was identified in both humans and animals with various renal diseases [9]. Additionally, the decrease in plasma KL has been suggested to be an indicator for DN progression in type 2 diabetic patients [10]. A growing number of evidences revealed that KL overexpression confers renoprotection. Haruna et al. [11] confirmed KL overexpression alleviated not only tubulointerstitial damage but also glomerulosclerosis. Wang et al. [12] displayed that KL overexpression weakened DN through ameliorating HG-triggered damage of glomerular endothelial cells. We and others also proved KL-conferred podocyte protection in DN [13–15]. Nevertheless, the molecular mechanism by which KL protects against podocyte injury in DN warrants further study.

The canonical transient receptor potential channel (TRPC) 6, a member of the TRPC family of nonselective Ca^2+^-permeable cation channels, has been implicated in the pathogenesis of glomerular diseases [16]. Gain-of-function mutations in TRPC6 cause hereditary familial focal segmental glomerulosclerosis [17, 18]. Moreover, TRPC6 is upregulated in several proteinuric kidney diseases including DN [19–21], which may be relevant to podocyte damage because Angiotensin II, reactive oxygen species (ROS), and other factors in the setting of DN directly activate TRPC6 activity and result in drastic increases in Ca^2+^ influx, causing podocyte actin cytoskeleton dynamics, hypertrophy, and foot process effacement [22–25]. Moreover, nonspecific overexpression of wide-type TRPC6 in mice leads to transient proteinuria [20], while TRPC6 deficiency protects podocytes and kidney function during the development of DN [26, 27]. These findings suggested that TRPC6 is an attractive treatment target of DN. Recently, the findings that KL exerts cardioprotection via the downregulation of TRPC6 pathways in the mouse heart [28] and that KL might mitigate proteinuria via targeting TRPC6 pathways in podocytes [29] raise the possibility that TRPC6 is a downstream target of KL in podocytes in DN. For that reason, herein, our study tested whether KL alleviated DN through inhibiting TRPC6 channel in HG-activated podocytes *in vitro* and diabetic *db/db* murine model *in vivo*.

## 2. Materials and Methods

### 2.1. Cell Culture and Treatment

The immortalized human podocyte cell line, offered by Prof. Niansong Wang (Shanghai Sixth People's Hospital, China), was cultured as depicted previously [30]. Briefly, undifferentiated podocytes were maintained at 33°C (the permissive temperature) in Roswell Park Memorial Institute 1640 (RPMI 1640) added with 10% Fatal Bovine Serum (FBS) and 1% insulin-transferrin-selenium (ITS, all from Thermo Fisher Scientific). For differentiation, cells were preserved under 37°C in the absence of ITS for 7 to 10 days to differentiate into mature podocytes. Mature podocytes were subjected to serum starvation in medium with 1% FBS prior to the exposure to 5 mM D-glucose (low glucose, LG) () or 30 mM D-glucose (HG) medium. For KL overexpression experiment, a recombination adenovirus vector involving murine KL full-length cDNA (Ad-KL) and the NC adenoviral vector involving green fluorescence protein (Ad-NC, CMV-MCS-SV40-EGFP) were purchased from Genechem (Shanghai, China). The differentiated podocytes were subjected to infection with Ad-KL or Ad-NC at a multiplicity of infection (MOI) of 20 for 24 h prior to cultivation with HG with/without 100 *μ*M TRPC6 activator flufenamic acid (FFA; Sigma-Aldrich, St Louis, MO, USA).

### 2.2. Animal Experiment

Ten week-old male diabetes C57BLKS/J-LepR (*db/db*) mice and littermate *db/m* mice were bought from the Model Animal Research Center of NU (Nanjing, China). All animals were kept under specific-pathogen-free (SPF) conditions under 25°C ± 1°C and moisture of 50% with a 12 h dark/light period and could eat and drink freely. The mouse experiment was accepted by the Ethical Committee of Putuo Hospital, Shanghai University of Traditional Chinese Medicine and completed as per the NIH guidance for the welfare of Lab animals (No. 8023, revised 1978). *Db/db* animals were stochastically separated into 3 groups (*n* = 6/group): the DN model group (*db/db* animals), DN+Ad-NC group (*db/db* animals with Ad-NC adenovirus injection), and DN+Ad-KL group (*db/db* mice with Ad-KL adenovirus injection). 50 *μ*l of Ad-NC or Ad-KL adenovirus (10^11^ PFU/ml) was inoculated into *db/db* animals via tail veins. D*b/m* animals were nondiabetic control group. Urine samples were collected with metabolic cages at d0, d7, d14, and d28 after adenovirus injection. Mouse urine albumin and creatinine contents were identified via commercially available kits (Biovision, Milpitas, CA, USA).

### 2.3. Histology

The collected renal samples were subjected to fixation with 4% paraformaldehyde (PFA), dehydration with ethanol, subjected to paraffin embedment, and sliced into 5 *μ*m slices. Then, the slices were stained with periodic acid Schiff (PAS) for morphological assessment. The mesangial matrix expansion was studied via Image Pro plus 6.0 (Media Cybernetics, America), and the semiquantitative score of glomerulosclerosis was identified via a five-grade approach depicted previously [31].

### 2.4. Transmission Electron Microscopy (TEM)

For ultrastructure test, 1 mm^3^ pieces of kidney cortex samples were subjected to fixation in 2.5% glutaric dialdehyde in 0.1 M phosphate buffering solution (pH = 7.4), postfixed in 1% osmium tetroxide, treated with dehydration in ethanol, and subjected to Spurr's epoxy resin embedment. Subsequently, ultrathin slices were obtained by ultramicrotome via a diamond cutter, double dyed in lead citrate and uranyl acetate, and studied via a Hitachi H7650 TEM imaging instrument. The width of podocyte foot process was measured and calculated as previously reported [32].

### 2.5. Immunofluorescence

Mouse kidney tissues were frozen in OCT (Thermo Fisher Scientific) and sectioned to 5 *μ*m thickness. The slices were subjected to fixation in prechilled acetone for 600 s under -20°C, blocked in 5% Bovine Serum Albumin (BSA) under RT for 60 min, and incubated at 4°C with the primary antibodies below: anti-KL antibody (Santa Cruz, CA, America), anti-Podocin antibody (Abcam, MA, USA), anti-WT-1 antibody (Abcam, MA, America), and anti-Synaptopodin antibody (Santa Cruz, CA, America). Fluorochrome-conjugated secondary antibodies were acquired from Jackson Immune Research Labs. 4′,6-diamidino-2-phenylindole (DAPI) was utilized for nucleus counterstaining.

### 2.6. Western Blot Analysis

Radio-Immunoprecipitation Assay (RIPA) lysis buffer (Beyotime, Shanghai, China) was applied to extract total proteins of kidneys or cultured podocytes. After the determination of protein concentrations, the proteins were separated via sodium dodecyl sulfate polyacrylamide gel electrophoresis (SDS-PAGE). Proteins were transferred onto a polyvinylidene fluoride (PVDF) membrane, blocked with 5% BSA for 60 min, and incubated with primary antibodies overnight at 4°C. After washing, secondary antibodies conjugated with horseradish peroxidase (HRP) were added for 1 h under room temperature. The visualization of the bands was realized via the chemiluminescent approach on GE Healthcare ImageQuant LAS 500 image formation instrument. The OD quantification was studied via Image J program (NIH, MD, America).

### 2.7. Quantitative Real-Time PCR (qRT-PCR)

Total RNA was abstracted from podocytes via TRIzol reagent (Invitrogen, CA, USA). 1 *μ*g of overall RNA was converted to cDNAs via a reverse transcript tool (Takara, Dalian, China). qRT-PCR was completed via ABI ViiA™ 7 Real-Time PCR System (Applied Biosystems) using TB Green Premix Ex Taq (Takara, Dalian, China). The PCR mix was incubated under 95°C for 10 min, before 40 cycles of 95°C for 15 sec and 60°C for 1 min. The sequences of primers utilized were presented below: human KL forward: 5′-CAGGACTATCTGCTGATGGACGA-3′ and human KL reverse: 5′-CTTTCAAGAGCTCGGCCATC-3′ and human *β*-actin forward: 5′-AAAGGGCAGGCAGGTTCTAT-3′ and human *β*-actin reverse: 5′-CTCCTTAATGTCACGCACGAT-3′. The comparative expression of KL was computed via 2^-△△Ct^ approach with *β*-actin as the internal control.

### 2.8. Phalloidin Staining

The cells grown on type-1 collagen (Invitrogen, America)-coated cover slips were subjected to fixation in 4% PFA in PBS for 15 min under 37°C, subjected to permeabilization for 5 min in 0.1% Triton X-100 in PBS, blocked for 30 min in PBS with 2% BSA, and incubated with TRITC-phalloidin (Molecular Probes, Invitrogen, Carlsbad, CA) for 20 min under room temperature without light. After washing with PBS, the nucleus was treated with counterstaining with DAPI. Fluorescent pictures were obtained using a laser scanning confocal microscopic device (LEICA DM IRB, Leica, Wetzlar, Germany). The fluorescent intensity of actin fibers was identified via the Image J software (NIH, MD, USA).

### 2.9. KL siRNA Transfection

Differentiated podocytes were subjected to transfection with KL siRNA (siKL) or negative control siRNA (siNC) (GenePharma, Shanghai, China) at an eventual content of 80 nM via Lipofectamine RNAiMAX Transfectional Reagent (Invitrogen, Carlsbad, CA, USA) as per the supplier's specification. The siKL sequence was presented below: sense: 5′-CCGAGAGCAUGAAGAAUAATT-3′ and antisense: 5′-UUAUUCUUCAUGCUCUCGGTT-3′. The siNC sequence was presented below: 5′-UUCUCCGAACGUGUCACGUTT-3′ and antisense: 5′-ACGUGACACGUUCGGAGAATT-3′. The siKL knockdown efficiency was evaluated via WB.

### 2.10. Statistics

Data are described as the mean ± SEM and were analyzed by GraphPad Prism program (GraphPad Software Inc., USA). A two-tailed Student's *t*-test was utilized for comparisons between 2 independent groups, and one-way ANOVA plus Tukey's posttest was employed to compare more independent groups. *P* < 0.05 had significance on statistics.

## 3. Results

### 3.1. KL Was Downregulated in HG-Stimulated Podocytes

**Figure 1:**
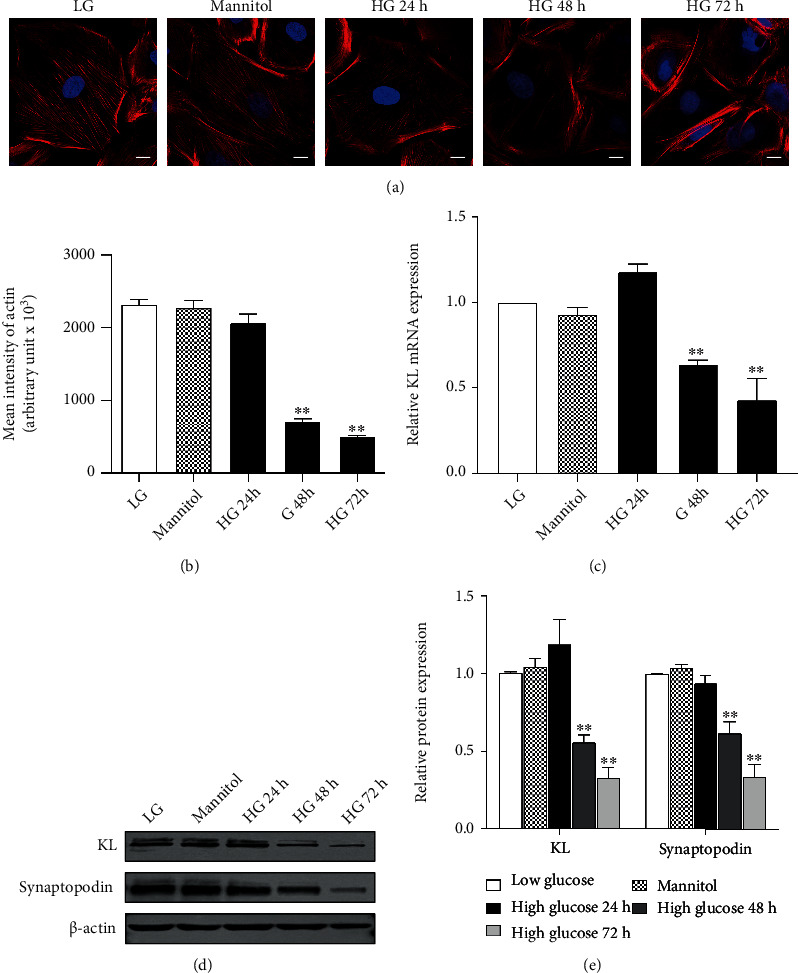
HG downregulated KL in podocytes. Podocytes were incubated with HG and collected at the indicated time points. (a) Typical images of phalloidin staining of human podocytes posterior to HG exposure. Scale bars, 10 *μ*m. (b) Quantified outcomes in (a). (c) The mRNA expression of KL at various time points. (d) Typical WB and (e) densitometric analyses of KL and Synaptopodin expression at different temporal points. *n* = 3. ^∗∗^*P* < 0.01 versus LG group.

Given that hyperglycemia contributes to DN pathogenesis, our study identified the impact of HG on KL expression in podocytes. As shown in Figures 1(a) and 1(b), podocyte cytoskeletons were remarkably disrupted by HG time-dependently as shown by TRITC-conjugated phalloidin staining. HG also reduced the expression levels of KL mRNAs and proteins time-dependently, paralleled by Synaptopodin downregulation (Figures 1(c)-1(e)).

### 3.2. Exogenous KL Protected against Podocyte Cytoskeletal Damage Induced by HG

**Figure 2:**
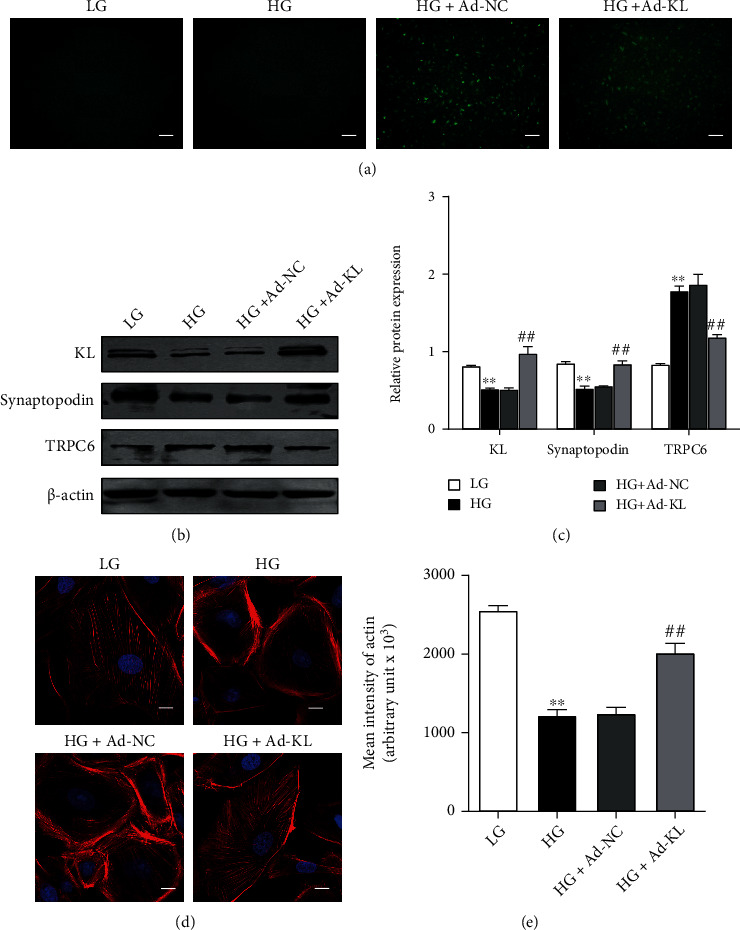
KL overexpression inhibited HG-induced podocyte injury. Podocytes were exposed to the adenovirus that expressed KL for 24 h and afterwards cultured with HG for extra 48 h. (a) The efficiency of Ad-NC and Ad-KL adenovirus infection examined by GFP fluorescence. (b) Representative western blots and (c) densitometric analyses of KL, Synaptopodin, and TRPC6 expression as aforementioned. (c) Typical images of phalloidin staining as aforementioned. Scale bars, 10 *μ*m. (d) Quantified outcomes in (c). *n* = 3. ^∗∗^*P* < 0.01 versus LG group. ^##^*P* < 0.01 versus HG group.

To test whether the restoration of KL expression could rescue podocyte cytoskeleton disruption induced by HG, we overexpressed KL in HG-cultured podocytes via Ad-KL infection. The efficiency of adenovirus infection was verified by fluorescence examination (Figure 2(a)). As presented by Figures 2(b) and 2(c), KL level in HG-stimulated podocytes was remarkably elevated after infection with Ad-KL, which was accompanied by an elevated expression of Synaptopodin and a decreased expression of TRPC6. TRITC-conjugated phalloidin staining showed that KL overexpression remarkably mitigated the disrupted F-actin stress fiber in podocytes induced by HG (Figures 2(d) and 2(e)).

### 3.3. KL Knockdown Damaged Podocyte Cytoskeleton

**Figure 3:**
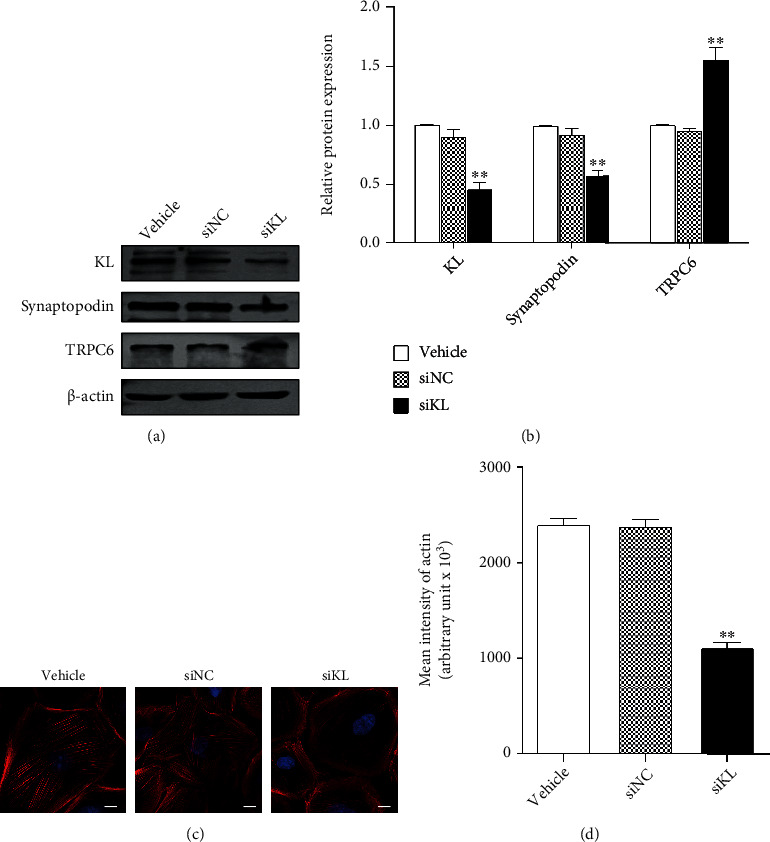
KL alleviated HG-induced podocyte injury via inhibiting TRPC6. Podocytes were exposed to the adenovirus that expressed KL for 24 h and afterwards cultured with HG for extra 48 h with or without 100 *μ*M FFA. (a) Typical WB and (b) densitometric analyses of TRPC6 and Synaptopodin expression as aforementioned. (c) Typical images of phalloidin staining as aforementioned. Scale bars, 10 *μ*m. (d) Quantification of the results in (c). *n* = 3. ^∗∗^*P* < 0.01 versus LG group. ^##^*P* < 0.01 versus HG group. ^&&^*P* < 0.01 versus HG+Ad-KL group.

To identity the role of KL downregulation in podocyte architecture, our study knocked down KL in cultured podocytes via KL-specific siRNA. KL knockdown resulted in the downregulation of Synaptopodin and the upregulation of TRPC6 (Figures 3(a) and 3(b)). In addition, the stress fiber quantity in podocytes was remarkably decreased via KL knockdown (Figures 3(c) and 3(d)).

### 3.4. KL Protected Podocyte Cytoskeleton via Inhibiting TRPC6

**Figure 4:**
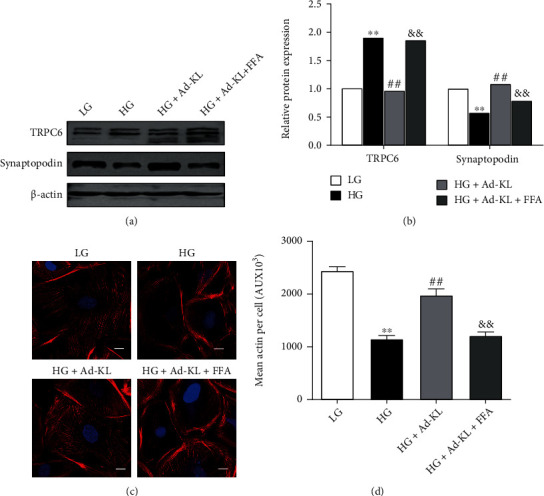
KL knockdown induced podocyte injury. (a) Typical WB and (b) densitometric analyses of KL, Synaptopodin, and TRPC6 expression in different groups. (c) Representative images of phalloidin staining in different groups. Scale bars, 10 *μ*m. (d) Quantification of the results in (c). Data were described as mean ± SEM. *n* = 3. ^∗∗^*P* < 0.01 versus vehicle group.

We sought to identify whether KL-conferred cytoskeletal protection was relevant to TRPC6. Western blot showed that cotreatment with TRPC6 activator FFA substantially abolished the upregulation of Synaptopodin expression conferred by KL overexpression in HG-cultured podocytes. Moreover, cotreatment with FFA significantly abrogated the protection of F-actin stress fibers conferred by KL overexpression (Figures 4(c) and 4(d)).

### 3.5. KL Was Downregulated in Glomerular Podocytes of Diabetic *db/db* Mice

**Figure 5:**
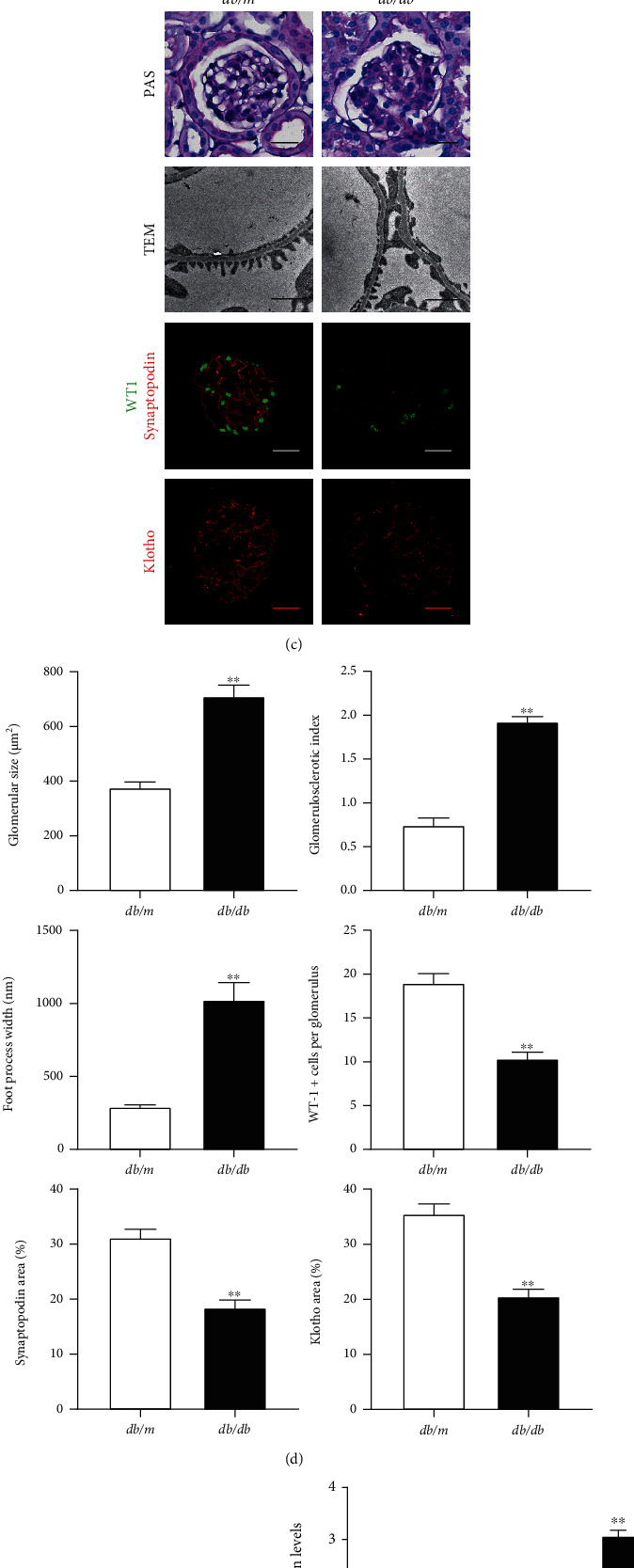
Decrease of KL expression in the glomeruli of *db/db* animals. (a) Glomerular KL expression levels in Ju CKD Glom data from Nephroseq. (b) Immunofluorescence staining unveiled that KL was colocalized with Podocin and Nephrin in podocytes. (c) Representative images of PAS, TEM, and immunofluorescence staining for WT-1, Synaptopodin, and KL in *db/m* and *db/db* animals. Scale bars for PAS, WT-1, Synaptopodin, and KL pictures, 10 *μ*m; Scale bars for TEM, 10 *μ*m. (d) Semiquantitation of glomerular size, glomerulosclerotic index, foot process width, quantity of podocytes positive for WT-1 per glomerulus, and Synaptopodin and KL expression areas in (c). (e) Typical WB and (f) densitometric analyses of KL, Synaptopodin, and TRPC6 expression in *db/m* and *db/db* animals. *n* = 3. ^∗∗^*P* < 0.01 versus *db/m* group.

The published transcriptomic data sets in the Nephroseq database showed that KL mRNA expressing was remarkably downregulated in the glomeruli of DN patients in contrast to normal samples (Figure 5(a)). Immunofluorescence staining of KL in mouse renal samples unveiled that KL existed in podocytes, as proved by colocalization of KL with Podocin and Nephrin, the biomarker proteins for podocytes (Figure 5(b)). As shown in Figures 5(c) and 5(d), 16-week-old *db/db* mice displayed massive glomerulus abnormalities, such as glomerular hypertrophy, glomerulosclerosis, and massive podocyte foot process effacement. Immunofluorescence staining revealed that podocyte KL expression was dramatically reduced accompanied by a reduction of podocyte cell-specific protein Synaptopodin and WT-1. Western blot analysis confirmed that KL and Synaptopodin were downregulated, and TRPC6 was upregulated in the kidney cortex of 16-week-old *db/db* animals (Figures 5(e) and 5(f)).

### 3.6. KL Overexpression Attenuated Podocyte Injury in *db/db* Mice

**Figure 6:**
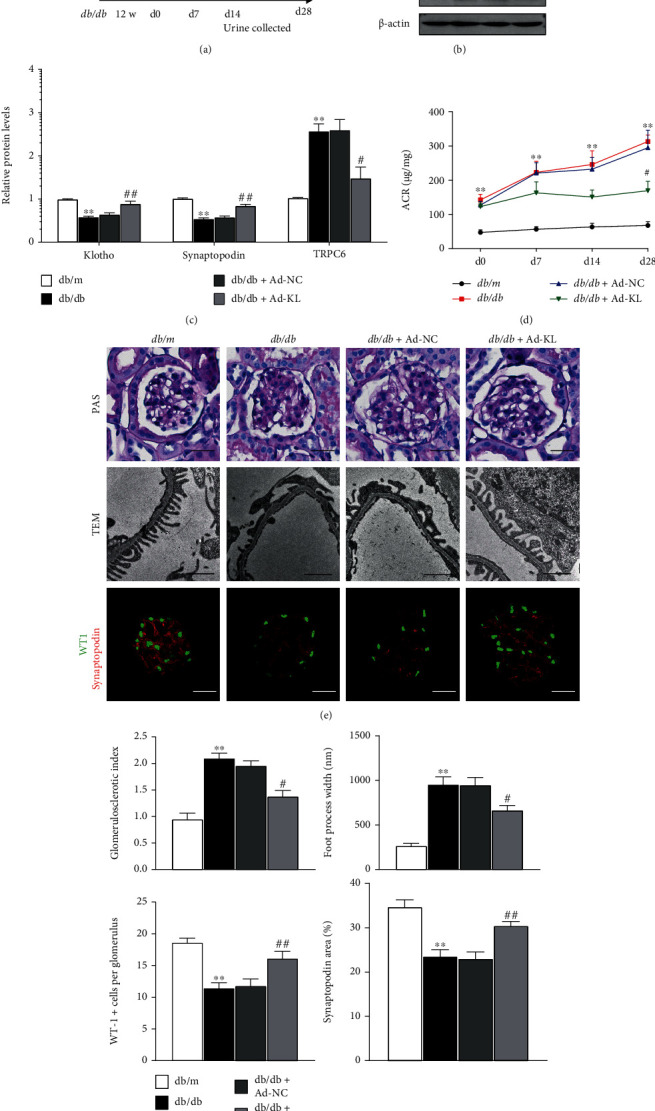
Renal KL overexpression inhibited TRPC6 and mitigated podocyte function disorder in *db/db* animals. (a) Schematic diagram of experiment process. 12-week-old *db/db* animals were inoculated with adenovirus expressing KL or negative control adenovirus via tail veins. Urine was harvested on 0 d, 7 d, 14 d, and 28 d postinjection with adenovirus, and renal samples were harvested on 28 d postinjection with adenovirus. (b) Representative western blots and (c) densitometric analyses of KL, Synaptopodin, and TRPC6 expression in the indicated groups. (d) Urinary ACR in the animals on days 0, 7, 14, and 28 postinjection with Ad-NC or Ad-KL. (e) Representative images of PAS, TEM, and immunofluorescence staining for WT-1 and Synaptopodin in the indicated groups. Scale bars for PAS staining, WT-1, and Synaptopodin pictures, 10 *μ*m; Scale bars for TEM, 10 *μ*m. (f) Semiquantitation of glomerulosclerotic index, foot process width, quantity of podocytes positive for WT-1 per glomerulus, and Synaptopodin expression region in (e). *n* = 4‐6. ^∗∗^*P* < 0.01 versus *db/m* group; ^##^*P* < 0.01 versus *db/db* group.

Finally, we examined the effects of KL overexpression on DN in *db/db* animals. We treated 12-week-old *db/db* animals with Ad-KL or Ad-NC via injection (Figure 6(a)). As presented by Figures 6(b) and 6(c), KL levels in the glomerular podocytes were remarkably elevated posterior to the injection of Ad-KL with a concomitant increase in Synaptopodin expression and a marked reduction in TRPC6 expression. The *db/db* animals displayed significant albuminuria, which was significantly alleviated by Ad-KL treatment (Figure 6(d)). Compared with the *db/db* animals exposed to Ad-NC, the animals challenged with Ad-KL showed ameliorated glomerulosclerosis, decreased podocyte foot process effacement, elevated Synaptopodin expression, and more cells positive for WT-1 in the kidneys (Figures 6(e) and 6(f)). These results indicated that KL could protect podocytes from injury and apoptosis, thereby improving glomerular filtration function and attenuating DN progression.

## 4. Discussion

Podocyte damage is pivotal for the etiopathogenesis of DN. TRPC6 overexpression- or activation-evoked intracellular Ca^2+^ increase contributes to the derangement of podocyte actin cytoskeleton leading to podocyte injury and dysfunction. In this study, we found that KL could inhibit HG-induced actin cytoskeleton disruption via downregulating TRPC6, whereas the knockdown of KL resulted in podocyte actin cytoskeleton damage *in vitro*. *In vivo*, Klotho overexpression suppressed TRPC6 expression and ameliorated podocyte injury in *db/db* animals. Those outcomes revealed that KL might exert renoprotective function through inhibiting TRPC6 channel.

Podocytes are special epitheliums on the outer aspect of the glomerulus capillaries with delicate interdigitating foot processes bridged by a greatly specialised junction, the slit diaphragm [33]. The highly dynamic foot processes contain an actin-based contractile apparatus that modulates the penetrability of the filtration barrier via variations in foot processes morphology [4]. Mutations of podocyte proteins that lead to actin cytoskeleton remodeling will disrupt the filtration barrier and result in kidney disease [33]. Therefore, the function of podocytes is largely based on their normal foot process structure, in particular on the maintenance of the actin cytoskeleton. Ca^2+^ dysregulation is pivotal for podocyte injury [34]. The elevation of Ca^2+^ in podocytes induces decomposition of Synaptopodin, an actin binding protein essential to podocyte completeness, and regulation of the Rho GTPase signal transmission which controls cytoskeleton kinetics and the morphology and migration of podocytes [34].

The TRPC pathway family comprises 7 members of nonselective Ca^2+^-permeation pathways. Amongst them, the expression of TRPC5 and TRPC6 is present in podocytes and is critical for modulating Ca^2+^ homeostasis and actin kinetics [5]. Large amount of researches have demonstrated that overactivity of TRPC5 or TRPC6 pathways induces actin cytoskeleton remodeling and albuminuria, and genetic silencing of either of them gives rise to protection [35–37]. TRPC6 is activated by hyperglycemia in renal resident cells in DN, including tubular cells, podocytes, and mesangial cells, while inhibiting TRPC6 expression or activity can delay DN progression [38–40]. Despite these findings, the upstream signaling regulating TRPC6 expression or activation has not been fully clarified.

KL is a membranous protein primarily generated in kidneys, which plays certain antiaging roles. KL deficiency has been implicated in glomerular pathophysiology [8]. A series of investigations have validated the protection potency of KL for podocytes in DN [12, 13, 41]. Nevertheless, whether and how KL directly protects podocytes are unknown. The association between KL and TRPC6 has been initially revealed by Xie et al. in 2012. They found that knockout of TRPC6 prevented stress-triggered heart remodelling in mice with insufficient KL [28]. Recently, another study provides evidence that KL suppressed TRPC6-mediated Ca^2+^ influx in cultured podocytes, decreased ATP-activated actin cytoskeleton rearrangement, and ameliorated TRPC6-induced albuminuria in mice [29]. KL upregulates TRPC6 via miR-30a to activate calcium/calcineurin signaling in PAN-stimulated podocytes [42]. Those discoveries revealed that TRPC6 is an underlying target of KL in podocytes in DN. Interestingly, we found that KL knockdown induced TRPC6 expression in podocytes. KL overexpression improved HG-evoked podocyte injury as demonstrated by actin cytoskeleton rearrangement and Synaptopodin downregulation and inhibited HG-induced TRPC6 expression. On the contrast, cotreatment with TRPC6 activator substantially abolished the favorable role of KL in HG-cultured podocytes. Consistently, KL overexpression improved the deteriorated renal function, the marked histopathology, and podocyte damage in *db/db* animals, with a concomitant downregulation of TRPC6. The *in vitro* and *in vivo* assays strongly suggested that KL might protect the actin cytoskeleton by inhibiting TRPC6 expression, thereby preventing podocyte injury and the progression of DN.

## 5. Conclusions

In summary, our research demonstrated that KL defended against hyperglycemia-induced podocyte injury in DN via suppressing TRPC6, which provided novel mechanistic insights for the role of KL in DN.

## Data Availability

The data used to support the findings of this study are included within the article.
